# Machine learning–based integrative analysis identifies CXCL13-driven tertiary lymphoid structures as favorable immune and prognostic features in osteosarcoma

**DOI:** 10.1007/s13402-026-01226-1

**Published:** 2026-05-16

**Authors:** Jie Jiang, Jiuhui Xu, Lu Xie, Yiyang Yu, Xin Sun, Xiaojiao Sun, Shen Yang, Huanmin Wang, Tingting Ren, Xiaodong Tang

**Affiliations:** 1 Department of Musculoskeletal Tumor, People’s Hospital, Peking University, Beijing, 100044 China; 2 Department of Surgical Oncology, Beijing Children’s Hospital, Capital Medical University, National Center for Children’s Health, Beijing, 100045 China; 3 MOE Key Laboratory of Major Diseases in Children, Beijing Children’s Hospital, Capital Medical University, National Center for Children’s Health, Beijing, 100045 China

**Keywords:** Osteosarcoma, Tertiary lymphoid structures, CXCL13, Tumor immune microenvironment, Machine learning

## Abstract

**Background:**

Osteosarcoma, an aggressive bone malignancy with limited response to immunotherapy, remains a major clinical challenge. Tertiary lymphoid structures (TLS), as organized ectopic lymphoid aggregates, play a pivotal role in modulating antitumor immune responses. However, their landscape, clinical significance, and molecular determinants in osteosarcoma remain largely unexplored.

**Methods:**

TLS were identified in osteosarcoma tissues by immunohistochemistry and multiplex immunofluorescence. Using transcriptomic data from the TARGET, GEO (GSE21257, GSE16091, GSE39055), and PKUPH cohorts, we developed a TLS-based prognostic index (TLSPI) through integrative machine-learning modeling. Functional enrichment, immune infiltration, and immunotherapy response prediction analyses were performed to characterize TLSPI-related biological features. The role of the key TLS-associated gene CXCL13 was validated through immunohistochemistry, single-cell transcriptomic profiling, and in vitro functional experiments.

**Results:**

TLS were detected in osteosarcoma and were significantly associated with improved overall survival (*p* = 0.024). Differential expression and enrichment analyses revealed that TLSPI-related genes were primarily involved in immune activation and antigen presentation pathways. Immune profiling revealed that the low-TLSPI group was characterized by greater immune infiltration and higher expression of immune checkpoint molecules, as well as lower TIDE scores, which indicated greater predicted sensitivity to immunotherapy. CXCL13 was identified as a key TLS-associated gene whose high expression correlated with improved survival. Immunohistochemical analysis confirmed the prognostic significance of CXCL13, while single-cell and functional assays demonstrated its association with B-cell activation within the osteosarcoma microenvironment.

**Conclusion:**

This study provides a comprehensive characterization of TLS in osteosarcoma and establishes a TLS-based prognostic index with potential clinical applicability. CXCL13 may serve as a critical mediator linking TLS formation and antitumor immunity, providing potential therapeutic implications for osteosarcoma immunotherapy.

**Supplementary Information:**

The online version contains supplementary material available at 10.1007/s13402-026-01226-1.

## Introduction

Osteosarcoma is the most common primary malignant bone tumor, primarily affecting children and adolescents [1–3]. Despite advances in multimodal therapy—including neoadjuvant chemotherapy, surgical resection, and adjuvant chemotherapy—the long-term survival for patients with recurrent or metastatic disease remains poor [4, 5]. Therapeutic progress has stagnated for decades, underscoring the urgent need for mechanism-based strategies to improve outcomes in this aggressive malignancy [6–8].

Immunotherapy has emerged as a promising approach by leveraging the host immune system to eliminate tumor cells. Strategies such as immune checkpoint blockade (ICB) [9], adoptive T-cell therapy [10], and chimeric antigen receptor (CAR) T-cell therapy [11] have demonstrated significant success in other malignancies. However, their efficacy in osteosarcoma remains limited. Increasing evidence suggests that osteosarcoma is an immunologically “cold” tumor, characterized by low tumor antigenicity, sparse cytotoxic T-cell infiltration, and an immunosuppressive microenvironment dominated by myeloid-derived suppressor cells (MDSCs), M2 tumor-associated macrophages (M2 TAMs), and cancer-associated fibroblasts (CAFs) [12–15]. Understanding this immune contexture is essential to identifying targets that could reinvigorate antitumor immunity.

Tertiary lymphoid structures (TLS) are ectopic lymphoid aggregates that develop in non-lymphoid tissues under chronic inflammatory conditions such as infection, autoimmunity, and cancer [16]. Structurally and functionally analogous to secondary lymphoid organs, TLS consist of B-cell follicles, T-cell zones enriched with dendritic cells, and high endothelial venules that provide specialized niches for antigen presentation and lymphocyte activation [17, 18]. Through these processes, TLS have emerged as key orchestrators of antitumor immunity.

Across diverse cancers—including melanoma [19], non–small cell lung cancer [20], and breast cancer [21]—TLS presence and maturation correlate with favorable prognosis and improved responsiveness to immune checkpoint blockade. In soft tissue sarcomas, TLS are associated with cytotoxic immune infiltration and proposed as a foundation for immune-related molecular classification [22]. However, their spatial distribution, composition, and biological significance in osteosarcoma remain largely undefined.

Among the molecular mediators of TLS organization, CXCL13 has been recognized as a pivotal chemokine driving B-cell recruitment and lymphoid neogenesis. Yet, its expression pattern, prognostic value, and functional role in osteosarcoma are poorly understood. In this study, we performed an integrative bioinformatics and pathological analysis to characterize the landscape and clinical significance of TLS in osteosarcoma. A TLS-based prognostic index (TLSPI) was established to evaluate patient outcomes and delineate TLS-associated immune and molecular features. Furthermore, CXCL13 was identified as a key TLS-related gene and validated through immunohistochemistry, single-cell transcriptomics, and in vitro assays. Collectively, our findings provide new insights into osteosarcoma immunobiology and highlight TLS and CXCL13 as potential biomarkers and therapeutic targets for translational cancer immunotherapy.

## Methods

### Data acquisition and preprocessing

Publicly available osteosarcoma datasets were curated under the following criteria: (1) histologically confirmed diagnosis, (2) complete survival information, and (3) survival time greater than 0. The TARGET osteosarcoma cohort (*n* = 84) and three GEO datasets (GSE21257, GSE16091, GSE39055) were retrieved and harmonized. GEO datasets were integrated into a combined GEO cohort (*n* = 123) after ComBat-based batch correction.

To expand the discovery cohort, osteosarcoma specimens were prospectively collected at Peking University People’s Hospital (PKUPH), including formalin-fixed paraffin-embedded (FFPE) tissues for immunohistochemistry and fresh-frozen tissues for RNA sequencing, forming the PKUPH transcriptomic cohort (*n* = 79). All cases were pathologically confirmed as primary osteosarcoma. Written informed consent was obtained from each participant, and the study protocol was approved by the Ethics Committee of PKUPH (IRB No. 2024PHB600-001). Clinical information (age, gender, and metastasis status) and follow-up data were systematically recorded. After cross-platform batch correction, a harmonized Meta cohort (*n* = 286) was constructed by integrating TARGET, combined GEO, and PKUPH datasets. Detailed characteristics are provided in Table S1.

### RNA sequencing of tissue samples

High-throughput mRNA sequencing was conducted by Berry Oncology. Total RNA was extracted using TRIzol Reagent (Thermo Fisher Scientific) and assessed for integrity by Agilent 2100 Bioanalyzer (RNA Integrity Number, RIN > 7.0). Libraries were prepared using the TruSeq Stranded mRNA Sample Preparation Kit (Illumina, RS-122-2101), including poly(A) selection, fragmentation, cDNA synthesis, and adapter ligation. Sequencing was performed on an Illumina NovaSeq 6000 platform with paired-end 2 × 150 bp reads (target depth approximately 10 million paired reads per sample).

Raw reads underwent quality control with FastQC, adapter and low-quality trimming using Trimmomatic, alignment to the human GRCh38 reference genome with STAR, and transcript quantification with RSEM to generate transcripts per million (TPM) values. TPM data were log₂-transformed, and batch effects were corrected using the ComBat algorithm.

### Immunohistochemistry (IHC)

FFPE Sect.  (4 μm) were baked at 65 °C, deparaffinized in xylene, and rehydrated through graded ethanol. Endogenous peroxidase activity was blocked with 3% hydrogen peroxide (H₂O₂) for 15 min, and nonspecific binding was blocked with 3% bovine serum albumin (BSA) for 1 h at room temperature. Primary antibodies against CD3 (Abcam, ab16669; 1:100) and CD20 (Abcam, ab78237; 1:100) were applied and incubated overnight at 4 °C. After PBS washes, slides were incubated with horseradish peroxidase (HRP)-conjugated secondary antibodies, dehydrated, mounted, and visualized under light microscopy.

### Multiplex immunofluorescence (mIF)

Adjacent FFPE Sect.  (4 μm) were deparaffinized, rehydrated, and subjected to antigen retrieval in citrate buffer (pH 6.0) at 95 °C for 15 min. Sections were blocked with 5% BSA (Sigma-Aldrich) at room temperature for 30 min. Primary antibodies from distinct host species—CD3 (HuaBio, HA720082; 1:1200), CD4 (HuaBio, ET1609-52; 1:1800), CD8 (Proteintech, 66868-1-Ig; 1:1500), CD20 (Sino Biological, 11007-MM06; 1:4000), and CD68 (HuaBio, EM1901-95; 1:500)—were sequentially incubated overnight at 4 °C. Corresponding fluorescent secondary antibodies were applied for 1 h in the dark, followed by nuclear counterstaining with DAPI (1 µg/mL, 5 min). Slides were mounted with anti-fade medium, and images were acquired on a Zeiss LSM 980 confocal microscope and analyzed using ZEN 3.3 software.

### TLSPI construction

The TLSPI was developed using the “Mime” R package [23], which provides an integrated, end-to-end framework for transcriptomics-based survival modeling by standardizing model training, evaluation, and visualization, thereby enabling fair benchmarking and improving reproducibility. A panel of 72 TLS-related genes was curated from recent literature [24, 25] (Table S2). In the TARGET cohort, prognostic candidate genes were first identified from this panel using univariate Cox regression (*p* < 0.05). These genes were then used as input features in Mime to evaluate 101 model combinations built from ten survival-learning algorithms (LASSO, GBM, RSF, plsRcox, stepwise Cox, SuperPC, ridge, survival-SVM, CoxBoost, and elastic net). The specific parameter settings for each algorithm are summarized in Table S3 [23]. To avoid information leakage, all feature selection, model comparison, and parameter tuning procedures were performed exclusively within the TARGET cohort using internal resampling procedures. The final TLSPI model was selected within the TARGET cohort based on internal discrimination and model parsimony, and the locked final model was subsequently applied unchanged to the GEO and PKUPH cohorts for independent external validation.

### Validation of prognostic performance

The predictive performance of TLSPI was validated across the TARGET, GEO, PKUPH, and Meta cohorts. Patients were divided into high- and low-risk groups according to the median TLSPI score. Overall survival differences were evaluated using Kaplan–Meier (KM) curves and log-rank tests, while predictive accuracy was quantified by time-dependent receiver operating characteristic (tROC) curves at 1-, 3-, and 5-year intervals, as well as by the C-index. In the PKUPH cohort, 2-, 3-, and 4-year time points were adopted because of a shorter follow-up duration. To assess robustness, stratified analyses were performed across subgroups defined by age, gender, and metastasis status.

### Comparison with published osteosarcoma signatures

To benchmark the prognostic performance of TLSPI against existing models in osteosarcoma, we conducted a PubMed-based systematic search and identified 90 previously published mRNA- or lncRNA-derived prognostic signatures (Table S4). Risk scores for each signature were computed using the original regression coefficients applied to Z-score-normalized gene expression profiles. Owing to gene naming updates and gene deletions in microarray datasets, only 36 published signatures could be reliably reconstructed in all four cohorts (TARGET, GEO, PKUPH, and Meta cohorts). Prognostic discrimination was evaluated using univariate Cox regression analysis, and predictive accuracy was compared using the C-index across these cohorts.

### Nomogram development and evaluation

Univariate and multivariate Cox regression analyses were performed to evaluate the independent prognostic value of TLSPI and clinical variables, including age, gender, and metastasis status. A nomogram was constructed to estimate 1-, 3-, and 5-year overall survival probabilities in patients with osteosarcoma. The predictive accuracy of the nomogram was assessed using calibration curves, and its clinical utility was further evaluated by decision curve analysis (DCA) in comparison with other clinical characteristics.

### Differential expression and functional enrichment analysis

Differentially expressed genes (DEGs) between high- and low-TLSPI groups were identified using the “limma” R package (|log₂ fold change| > 1, FDR < 0.05). Functional enrichment analyses of DEGs were conducted for Gene Ontology (GO) terms and Kyoto Encyclopedia of Genes and Genomes (KEGG) pathways using the “clusterProfiler” package. To further explore the biological pathways differentially enriched between the two groups, Gene Set Enrichment Analysis (GSEA) was performed to identify hallmark gene sets (|NES| > 1, *p* < 0.05).

### Immune infiltration analysis

Tumor immune activity was assessed using the “ESTIMATE” R package to calculate stromal, immune, and ESTIMATE scores for each patient [26]. Immune cell infiltration was estimated using CIBERSORT and cross-validated with xCell, TIMER, quanTIseq, MCP-counter, IPS, and EPIC via the “IOBR” package [27]. Gene Set Variation Analysis (GSVA) was performed to compare immune-related functional pathways between high- and low-TLSPI groups. In addition, correlations between TLSPI and immune checkpoint genes (IDO1, LAG3, CTLA4, PD-L2, TIGIT, PD-1, LAIR1, CD276, PD-L1, HAVCR2, BTLA, LGALS9, and CD200R1) were systematically investigated.

### Immunotherapy response prediction and drug sensitivity analysis

Potential responses to immunotherapy were predicted using the Tumor Immune Dysfunction and Exclusion (TIDE) algorithm (https://tide.dfci.harvard.edu/) [28], where higher TIDE scores indicate greater immune evasion and reduced immunotherapy efficacy. The prognostic value of TLSPI was further validated in two independent immunotherapy cohorts (GSE100797 and GSE35640). Drug sensitivity analyses were performed with the oncoPredict R package [29] using the Genomics of Drug Sensitivity in Cancer datasets (GDSC1 and GDSC2). Predicted half-maximal inhibitory concentrations (IC50) for the tested agents were compared between TLSPI-defined subgroups.

### Single-cell RNA sequencing analysis

The Tumor Immune Single Cell Center 2 (TISCH2; http://tisch.comp-genomics.org) [30], an online database that integrates 190 single-cell transcriptomic profiles across more than 50 cancer types, was used to perform single-cell RNA sequencing analysis. In this study, the GSE162454 dataset, comprising six primary osteosarcoma samples, was examined via TISCH2 to explore cell clustering, cell type annotation, and the expression patterns of TLS-related genes visualized on UMAP plots.

### B cell isolation and culture

Human B cells were isolated from peripheral blood mononuclear cells (PBMCs) obtained from anonymous healthy donors under a separately approved protocol (IRB No. 2025PHB112-001) with written informed consent. CD19 microbeads (Stemcell Technologies, Cat. 17954) were used as per the manufacturer’s instructions. The isolated cells were cultured in RPMI 1640 medium with 10% fetal bovine serum (FBS) at 37 °C in 5% CO₂ for 72 h.

### Transwell migration assay

B cells (1 × 10⁴ cells per well) were seeded into the upper chambers of Transwell inserts containing serum-free medium. The lower chambers were filled with 500 µL RPMI 1640 medium supplemented with 10% FBS and 500 ng/mL CXCL13 (PeproTech, Cat. 300-47-5UG) as a chemoattractant. After incubation at 37 °C for 12 h, migrated cells on the lower surface of the membrane were fixed with 4% paraformaldehyde and stained with crystal violet for 15 min.

### RNA sequencing of B cells

Total RNA was extracted from B cells stimulated with 500 ng/mL CXCL13 for 72 h. mRNA was purified and sequencing libraries were prepared following standard protocols. Sequencing was performed on the Illumina NovaSeq 6000 platform (Beijing Genomics Institute). Raw reads were quality-controlled using Fastp, aligned to the human reference genome (GRCh38) with STAR, and quantified at the gene level using FeatureCounts.

### Flow cytometry

B lymphocytes were stimulated with 500 ng/mL CXCL13 for 72 h and stained with anti-CD80 (Proteintech, Cat. 65083), anti-CD86 (Proteintech, Cat. 65165), and anti-MHC-II (Proteintech, Cat. 65218). Apoptosis was assessed using an Annexin V-FITC/PI detection kit (Beyotime Biotechnology, C1062S) following the manufacturer’s protocol. Flow-cytometric analysis was performed on a BD Accuri C6 Plus cytometer, and data were processed using FlowJo software (v10.8.1, BD Biosciences). Gating strategies were applied to identify single, live cells and relevant subpopulations.

### Statistical analysis

All analyses were performed in R (v4.2.0). Between-group differences were assessed using Student’s t-test or Wilcoxon rank-sum test, as appropriate. Spearman’s rank correlation was used to evaluate associations between TLSPI and immune-related features. Survival differences were analyzed by KM curves with log-rank tests, and hazard ratios (HRs) were estimated using Cox proportional hazards regression. Unless otherwise specified, data are presented as mean ± standard deviation (SD), and error bars indicate SD from independent biological replicates (n is provided in the corresponding figure legends). All p values were two-sided, and *p* < 0.05 was considered statistically significant (*p < 0.05; **p < 0.01; ***p < 0.001).

## Results

### Histological identification and prognostic significance of TLS in osteosarcoma

**Fig. 1:**
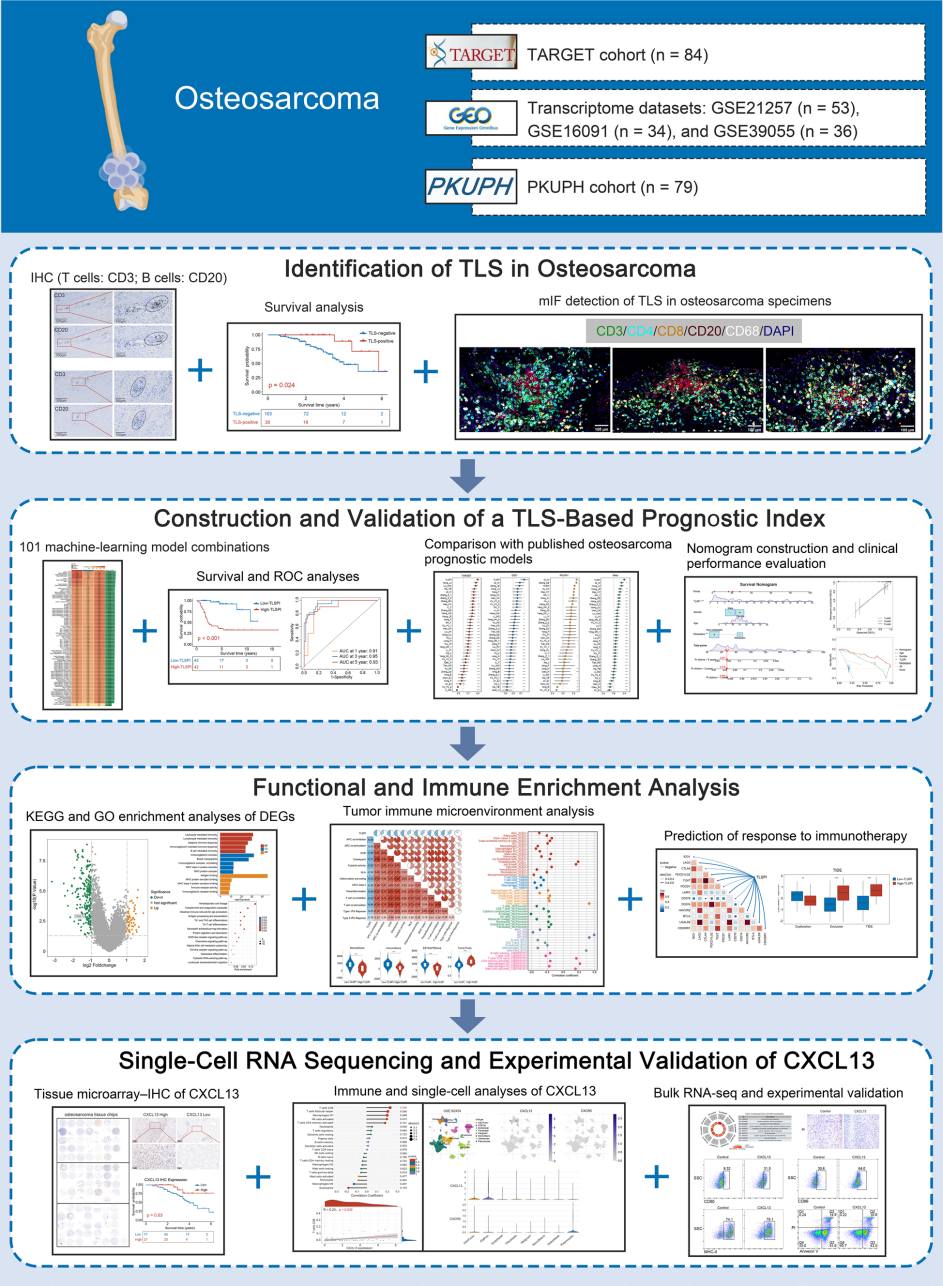
Overview of the study design and analytical workflow illustrating the main experimental and computational steps

**Fig. 2:**
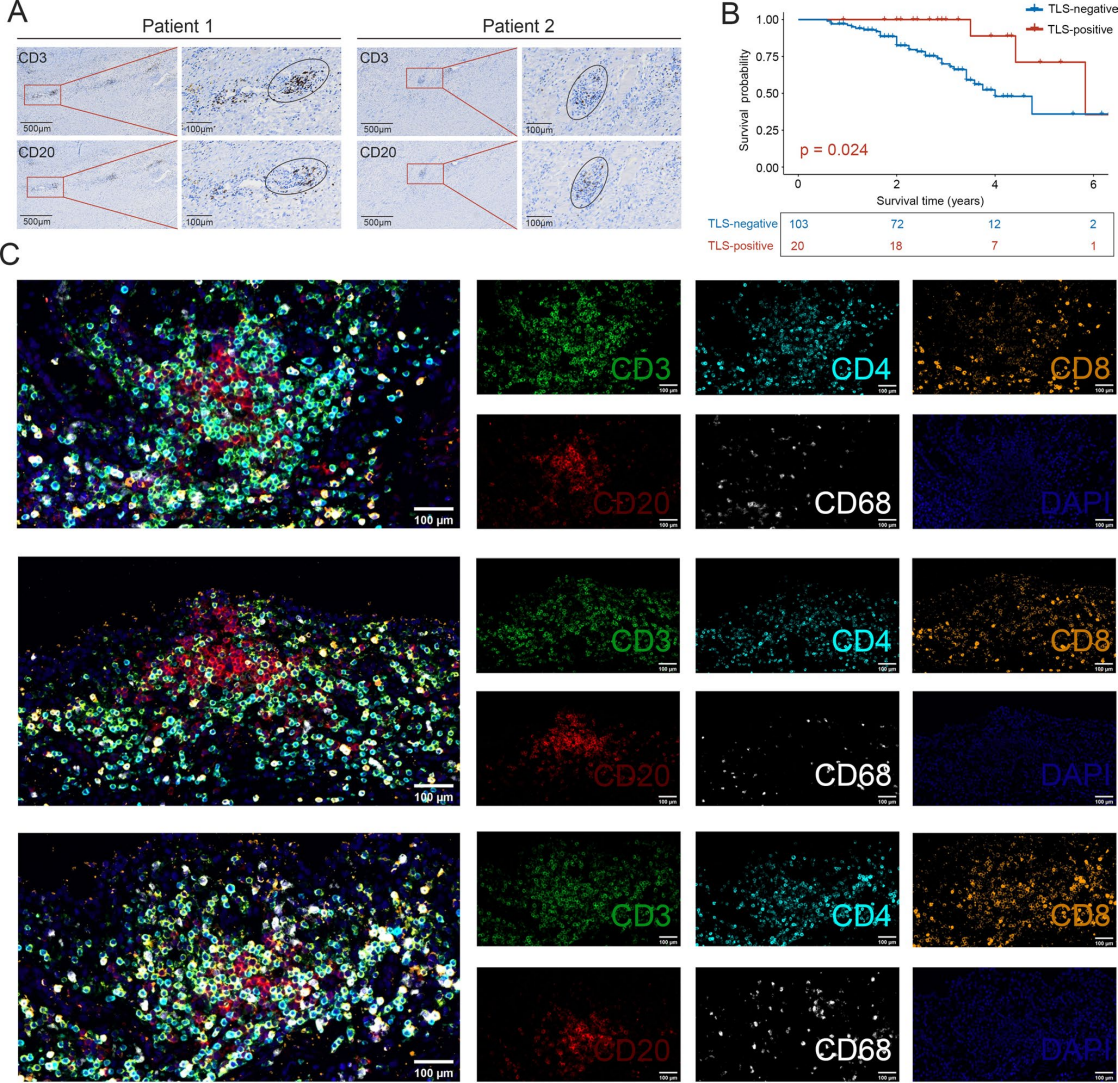
Identification of TLS in osteosarcoma. (**A**) Representative IHC staining showing CD3⁺ T-cell and CD20⁺ B-cell clusters in osteosarcoma tissue sections. Circled areas indicate TLS-like aggregates. (**B**) KM survival curves comparing overall survival between TLS-positive and TLS-negative patient groups. (**C**) mIF analysis of TLS composition in serial osteosarcoma specimens using CD3, CD4, CD8, CD20, and CD68 antibodies, revealing organized immune-cell aggregates consistent with TLS-like features

The overall methodological workflow of this study is summarized in Fig. 1. Histological examination identified TLS-like structures in osteosarcoma tissues, characterized by the co-localization of CD3⁺ T-cell zones and CD20⁺ B-cell follicles within the tumor stroma (Fig. 2A). Among 123 IHC-assessed osteosarcoma specimens, TLS-like formations were detected in 20 cases (16.3%). KM survival analysis revealed that patients harboring TLS-like structures had significantly longer overall survival than those without such features (*p* = 0.024; Fig. 2B).

Multiplex immunofluorescence further confirmed the presence of TLS in osteosarcoma, revealing spatially organized aggregates of CD3⁺, CD4⁺, and CD8⁺ T cells, CD20⁺ B cells, and CD68⁺ macrophages. These TLS displayed well-compartmentalized B-cell follicles and T-cell zones, closely recapitulating canonical TLS architecture (Fig. 2C), consistent with previously described TLS features [31]. Collectively, these findings provide histological and spatial evidence for TLS formation in osteosarcoma and suggest that TLS presence represents a favorable prognostic indicator within the tumor immune microenvironment (TIME).

### Establishment and validation of TLSPI

**Fig. 3:**
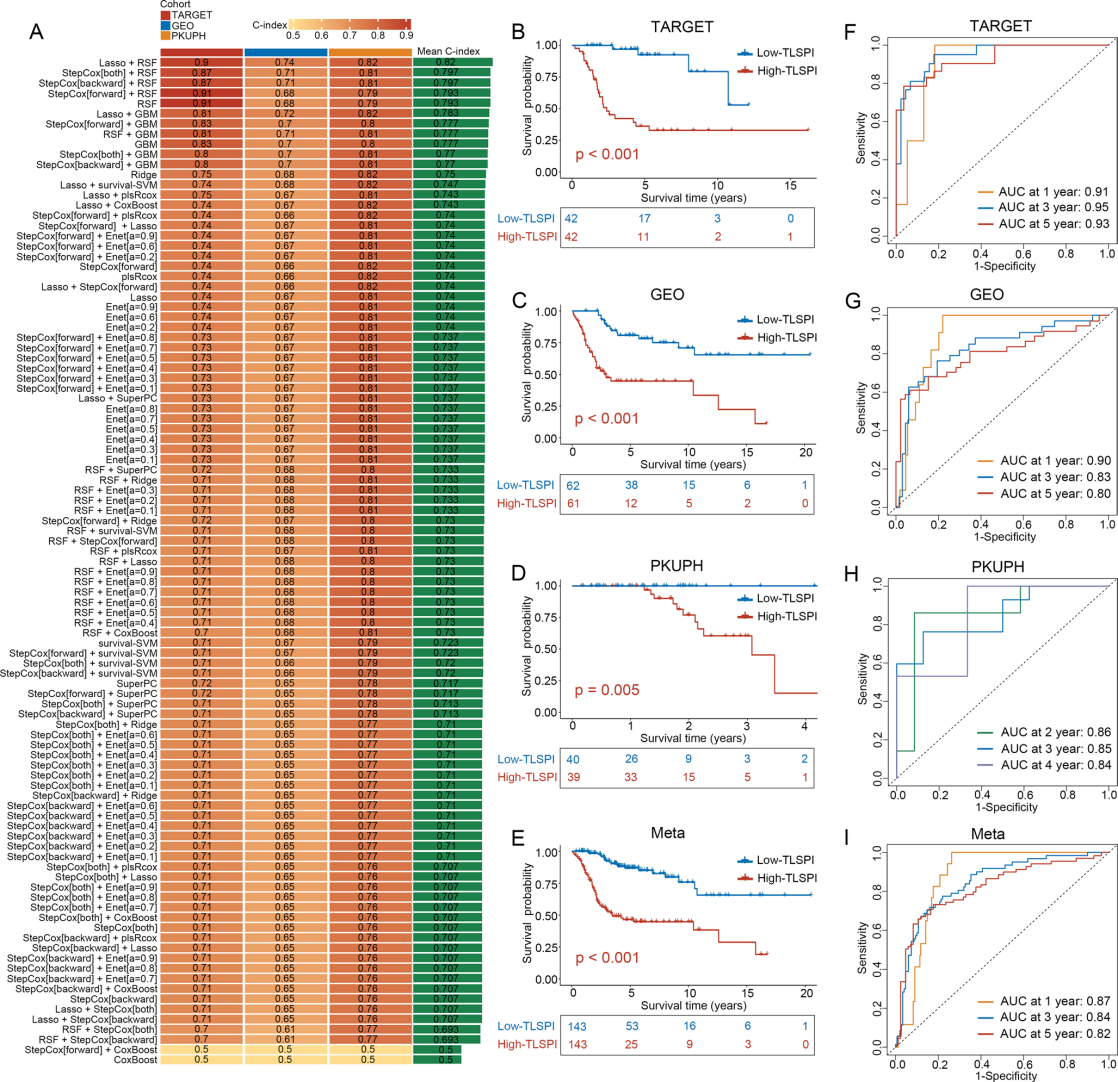
Development and validation of TLSPI. (**A**) Comparative evaluation of 101 machine-learning model combinations based on the C-index in the training (TARGET) and validation (GEO, PKUPH) cohorts. (**B**–**E**) KM survival curves showing overall survival differences between TLSPI-defined subgroups in four independent cohorts. (**F**–**I**) tROC curves illustrating predictive accuracy for 1-, 3-, and 5-year overall survival in TARGET, GEO, and Meta cohorts, and for 2-, 3-, and 4-year survival in PKUPH

From the 72 TLS-related genes curated from the literature, univariate Cox regression analysis in the TARGET cohort identified 17 prognostic candidates (*p* < 0.05; Table S5). These genes were then used as input features for 101 model combinations evaluated across ten survival-learning algorithms. Among the candidate pipelines, LASSO + RSF was selected as the final model based on internal discrimination and model parsimony (Fig. 3A). In this optimal pipeline, LASSO (alpha = 1; nfold = 10; family = “cox”) retained three genes (CXCL13, IL2RA, and STAT5A). The RSF model was then trained using these features (ntree = 1000; nodesize = 5; splitrule = “logrank”; importance = TRUE; proximity = TRUE; forest = TRUE), and TLSPI was defined as the RSF-predicted individual risk score. Patients stratified by the median TLSPI showed significantly different outcomes, with higher mortality in the high-TLSPI group (Fig. S1).

Survival analyses confirmed the consistent prognostic performance of TLSPI across multiple datasets. KM curves demonstrated significantly better outcomes in the low-TLSPI subgroup in all cohorts (TARGET *p* < 0.001; GEO *p* < 0.001; PKUPH *p* = 0.005; Meta cohort *p* < 0.001; Fig. 3B–E). In addition, tROC analysis validated the predictive performance of TLSPI, with area under the curve (AUC) values for 1-, 3-, and 5-year overall survival of 0.91, 0.95, and 0.93 in TARGET; 0.90, 0.83, and 0.80 in GEO; 0.86, 0.85, and 0.84 for 2-, 3-, and 4-year survival in PKUPH; and 0.87, 0.84, and 0.82 in the Meta cohort (Fig. 3F–I). To further assess robustness across heterogeneous clinical contexts, stratified survival analyses by age, gender, and metastasis status confirmed that TLSPI retained significant prognostic value within all subgroups (Fig. S2), demonstrating its stability and generalizability across patient populations.

### Comparison of TLSPI with published prognostic signatures

**Fig. 4:**
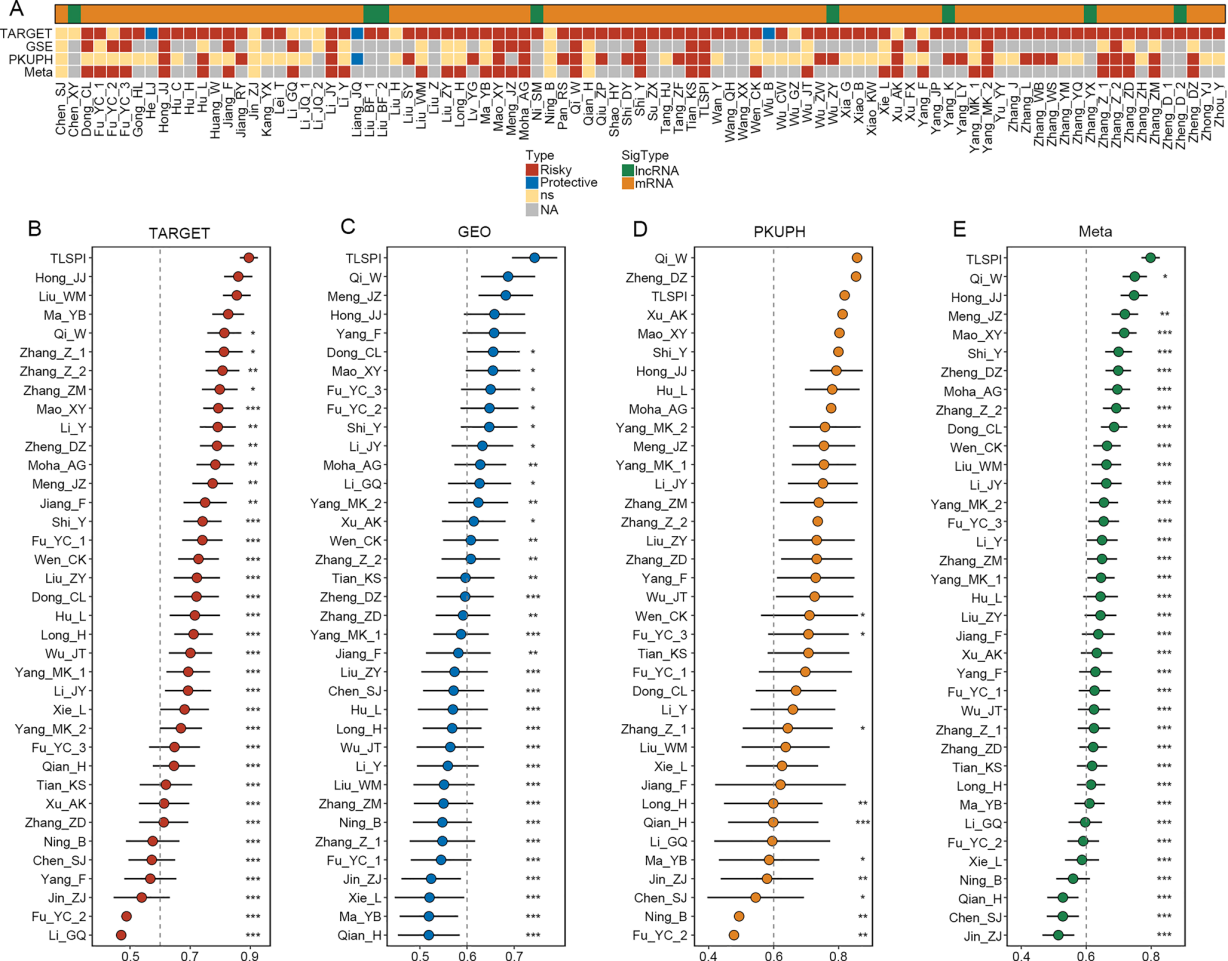
Comparative evaluation of TLSPI and published osteosarcoma prognostic signatures. (**A**) Univariate Cox regression results for TLSPI and published signatures in the TARGET, GEO, PKUPH, and Meta cohorts. (**B**–**E**) Comparison of C-index values between TLSPI and other evaluable models across the four datasets

The predictive performance of TLSPI was benchmarked against 90 previously published osteosarcoma signatures applicable to at least one cohort. Because of gene-set inconsistencies and incomplete annotations, 36 signatures were ultimately evaluable across datasets. Among these, 10 models achieved statistical significance in all datasets (Fig. 4A). Notably, TLSPI consistently outperformed other signatures in the TARGET, GEO, and Meta cohorts, and retained robust prognostic discrimination in the PKUPH cohort (Fig. 4B–E), highlighting its superior stability and generalizability across independent datasets.

### Prognostic independence and construction of a TLSPI-based nomogram

**Fig. 5:**
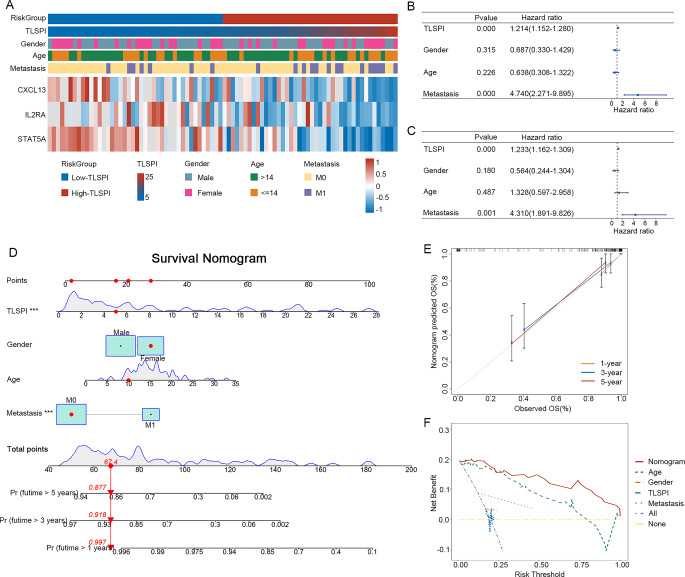
Clinical utility of TLSPI. (**A**) Heatmap showing clinicopathological characteristics and expression levels of the three TLSPI genes in the TARGET cohort. (**B**, **C**) Univariate (**B**) and multivariate (**C**) Cox regression analyses evaluating the independent prognostic value of TLSPI and clinical parameters. (**D**) Nomogram integrating TLSPI and clinical variables to predict 1-, 3-, and 5-year overall survival. (**E**) Calibration plots demonstrating agreement between predicted and observed survival at 1-, 3-, and 5-year time points. (**F**) DCA comparing the net clinical benefit of the nomogram with that of individual clinicopathological factors

Clinicopathological characteristics and expression levels of TLSPI component genes were visualized via a heatmap, revealing clear differences between risk subgroups in the TARGET cohort (Fig. 5A). Both univariate and multivariate Cox regression analyses confirmed TLSPI as an independent prognostic factor (HR = 1.214, 95% CI 1.152–1.280, *p* < 0.001; multivariate HR = 1.233, 95% CI 1.162–1.309, *p* < 0.001; Fig. 5B–C), independent of clinical parameters such as age, gender, and metastatic status.

To facilitate individualized survival prediction, a nomogram integrating TLSPI with key clinical variables was developed to estimate 1-, 3-, and 5-year overall survival probabilities (Fig. 5D). Calibration plots demonstrated excellent agreement between predicted and observed outcomes (Fig. 5E), while DCA indicated that the nomogram provided a greater net clinical benefit than any single clinical variable (Fig. 5F).

### Biological function analysis of TLSPI

**Fig. 6:**
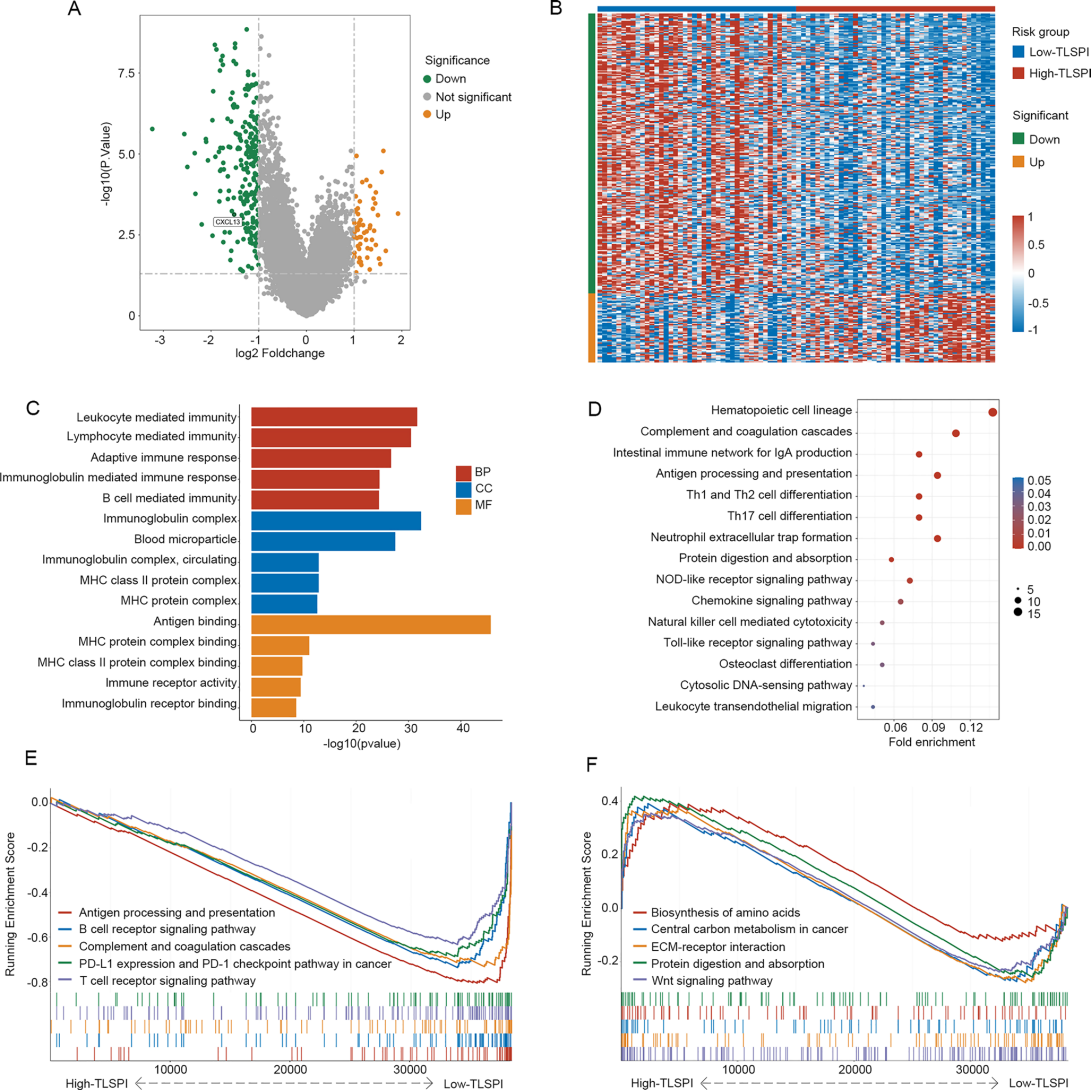
DEGs detection and functional enrichment analysis. (**A**) Volcano plot displaying DEGs in the high- versus low-TLSPI groups. (**B**) Heatmap showing expression patterns of DEGs across the two TLSPI-defined subgroups. (**C**, **D**) GO (**C**) and KEGG (**D**) enrichment analyses of DEGs. (**E**, **F**) GSEA illustrating differentially enriched pathways between different patient subgroups

To explore biological pathways associated with TLSPI, gene expression profiles were compared between low- and high-TLSPI subgroups. A total of 283 DEGs were identified in the high-TLSPI group relative to the low-TLSPI group, including 56 upregulated and 227 downregulated genes (Fig. 6A–B). CXCL13 was notably overexpressed in the low-TLSPI group. GO enrichment analysis indicated that these DEGs were mainly involved in immune-related biological processes, such as B-cell-mediated and leukocyte-mediated immunity, and immunoglobulin responses. In the cellular component category, enrichment was observed in the immunoglobulin and MHC protein complexes, while molecular function terms highlighted antigen binding, MHC interaction, and immunoglobulin receptor binding (Fig. 6C). KEGG pathway analysis further revealed enrichment of immune-related pathways, including antigen processing and presentation, Th1/Th2, and Th17 cell differentiation (Fig. 6D).

GSEA identified distinct activation patterns between TLSPI-defined subgroups: tumors with low TLSPI exhibited enrichment of immune activation pathways, including T- and B-cell receptor signaling and PD-1/PD-L1 checkpoint signaling (Fig. 6E), whereas those with high TLSPI showed upregulation of Wnt signaling and central carbon metabolism pathways (Fig. 6F). Collectively, these results suggest that TLSPI is tightly linked to immune activation status in osteosarcoma, providing a biological rationale for subsequent immune correlation analyses.

### Immune cell infiltration analysis

**Fig. 7:**
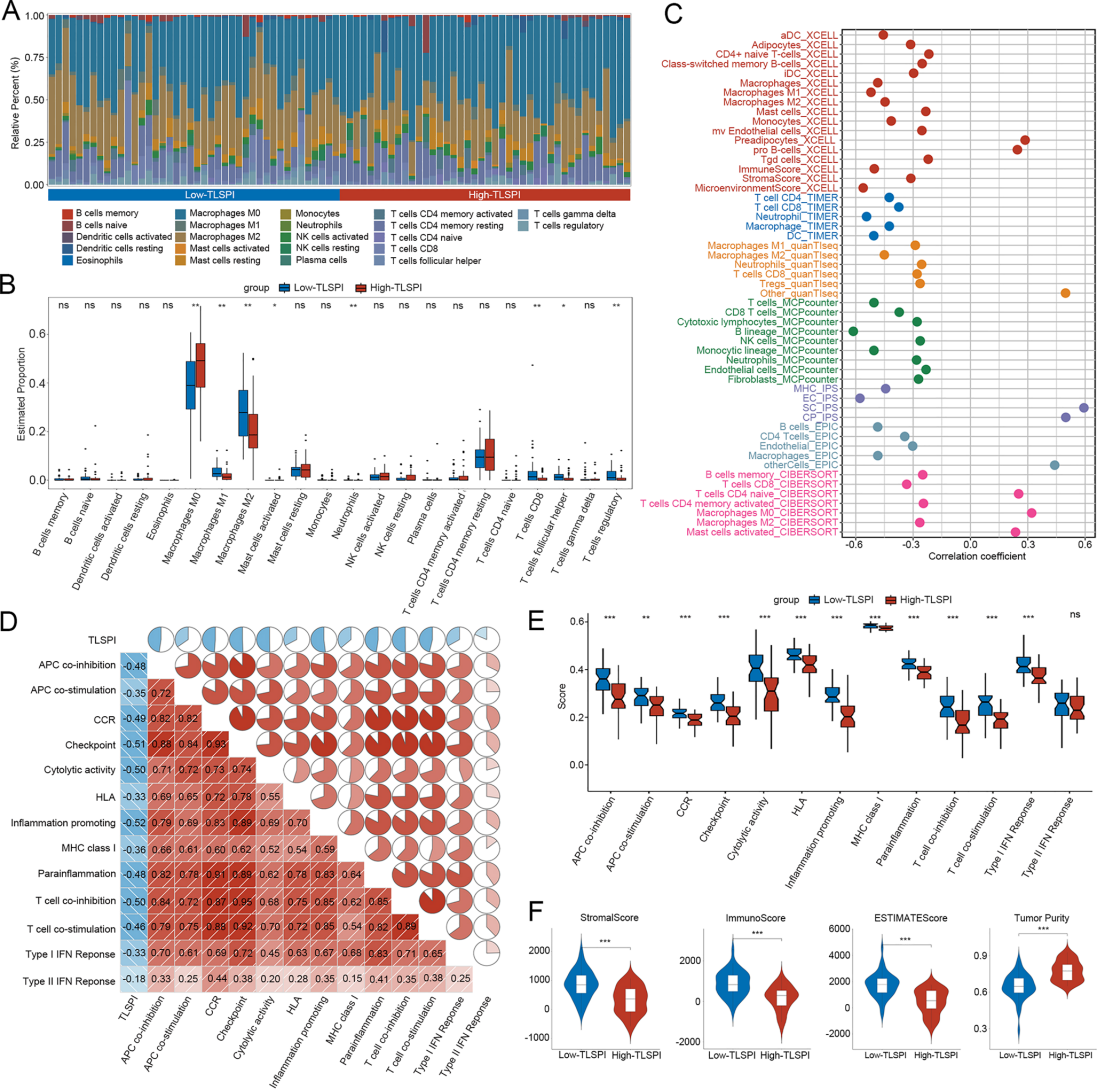
Association of TLSPI with TIME. (**A**) Composition of 22 immune cell types estimated by CIBERSORT analysis. (**B**) Differential abundance of immune cell subsets between the high- and low-TLSPI groups. (**C**) Bubble plot depicting immune cell infiltration patterns across TLSPI-defined subgroups. (**D**) Correlogram showing correlations between TLSPI and immune-related functional signatures, where red and blue indicate positive and negative correlations, respectively. (**E**) Differential GSVA scores representing immune-related functional signatures in the two subgroups. (**F**) Comparison of stromal, immune, and ESTIMATE scores, as well as tumor purity, between high- and low-TLSPI groups

To further characterize the TIME in osteosarcoma, CIBERSORT analysis was performed and revealed that macrophages (M0 and M2) constituted the predominant infiltrating immune-cell populations (Fig. 7A; Table S6). Significant differences in macrophage and T cell proportions were observed between the high- and low-TLSPI subgroups, with CD8⁺ T cell infiltration markedly higher in the low-TLSPI group (Fig. 7B). The association between TLSPI and immune infiltration was further assessed using seven independent deconvolution algorithms, which consistently revealed negative correlations between TLSPI and cytotoxic lymphocyte populations (CD8⁺ T cells and NK cells) as well as B-cell abundance (Fig. 7C).

Given the central role of immune infiltration in shaping immune activity, immune-related functional signatures were further evaluated using GSVA. The analysis confirmed an inverse relationship between TLSPI and multiple immune activation signatures—including checkpoint activity, inflammation-promoting pathways, T-cell co-stimulation, among others (Fig. 7D)—with significantly higher immune function scores observed in the low-TLSPI group (Fig. 7E). Furthermore, tumor purity, stromal, immune, and ESTIMATE scores were calculated using the ESTIMATE algorithm, revealing that patients with low TLSPI exhibited significantly higher stromal, immune, and ESTIMATE scores, along with reduced tumor purity (Fig. 7F; Table S7).

### Immunotherapy efficacy based on TLSPI risk signature

**Fig. 8:**
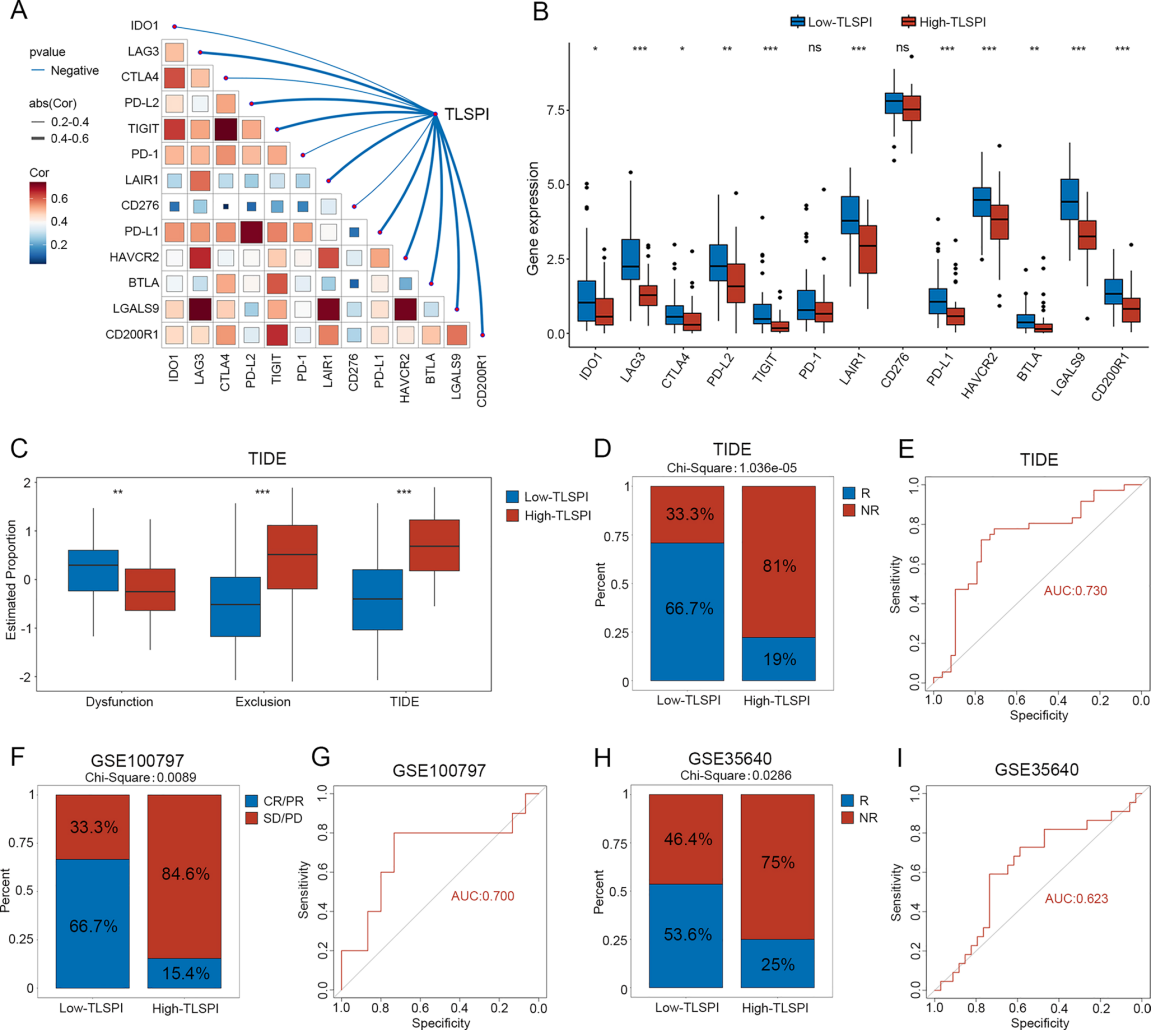
TLSPI-associated immunotherapy response and drug sensitivity analyses. (**A**) Correlation between TLSPI and common immune checkpoint molecules. (**B**) Differential expression of immune checkpoint molecules between high- and low-TLSPI subgroups. (**C**) Comparison of T-cell dysfunction, T-cell exclusion, and TIDE scores between low- and high-TLSPI groups. (**D**, **E**) TIDE-predicted immunotherapy response and ROC analysis based on the TARGET cohort. (**F**–**I**) Validation of TLSPI-associated response patterns in external immunotherapy datasets, including GSE100797 (**F**, **G**) and GSE35640 (**H**, **I**). PD, progressive disease; SD, stable disease; PR, partial response; CR, complete response; R, response; NR, non-response

Given the therapeutic relevance of immune checkpoints, their association with TLSPI was further examined. TLSPI exhibited negative correlations with the expression of multiple immune checkpoint genes (Fig. 8A). Consistently, the low-TLSPI group displayed higher expression levels of checkpoint molecules such as LAG3, CTLA4, and PD-L1 compared with the high-TLSPI group (Fig. 8B). Within the TARGET cohort, application of the TIDE framework revealed that low-TLSPI patients had higher T-cell dysfunction but lower T-cell exclusion and overall TIDE scores (Fig. 8C), indicating a more immune-active phenotype and enhanced predicted responsiveness to immunotherapy. This trend was further reflected in a higher proportion of responders and favorable predictive performance (AUC = 0.730; Fig. 8D–E).

Consistent results were observed in external immunotherapy datasets. In GSE100797 and GSE35640, low-TLSPI patients similarly showed a higher proportion of responders compared with high-TLSPI patients (Fig. 8F, H). Corresponding ROC analyses demonstrated robust predictive performance, with AUC values of 0.700 and 0.623, respectively (Fig. 8G, I). Together, these findings suggest that osteosarcoma patients with low TLSPI are more likely to benefit from immunotherapeutic interventions.

### Identification of candidate drugs for osteosarcoma subgroups

**Fig. 9:**
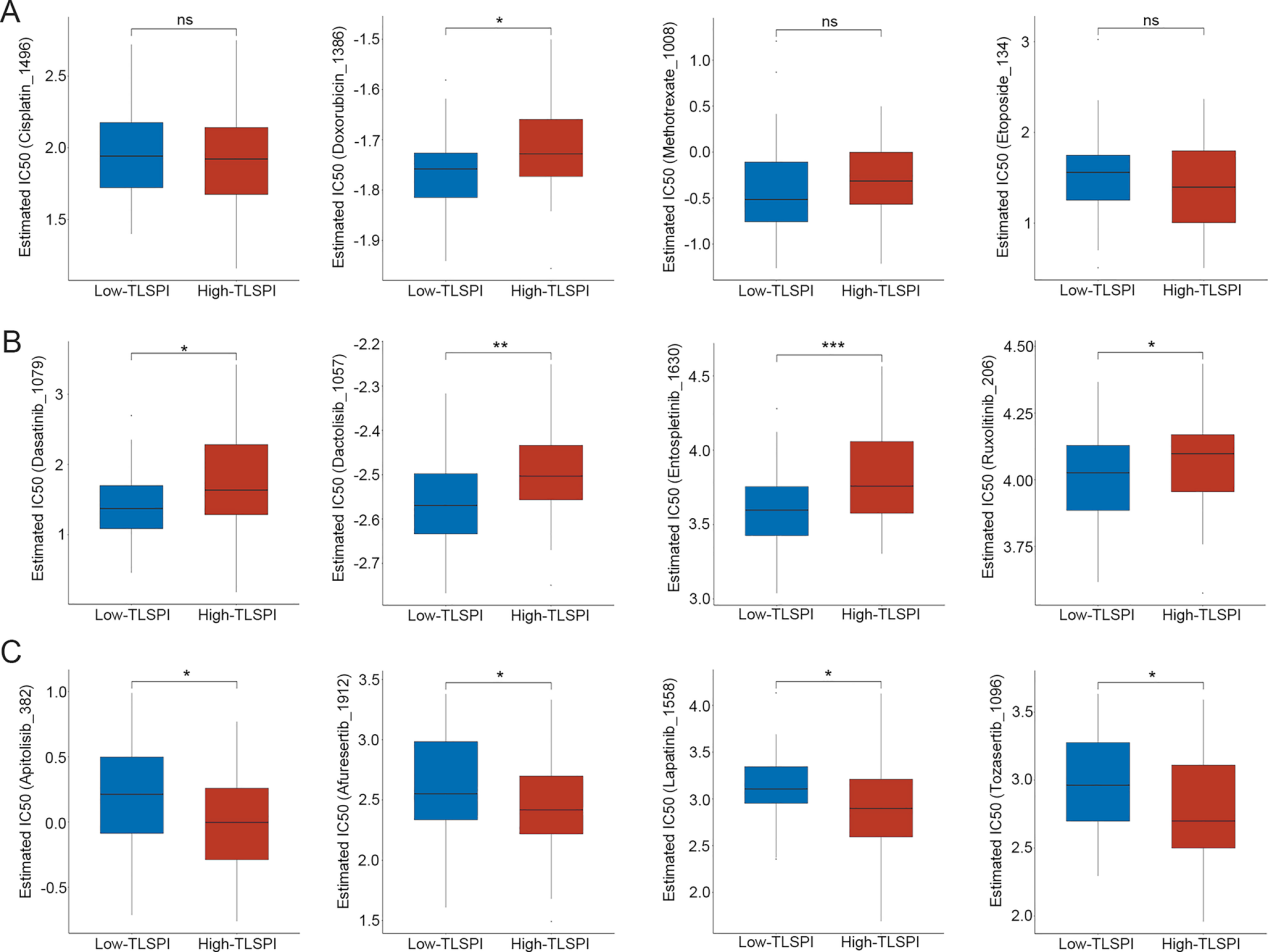
Predicted IC50 by TLSPI subgroup in osteosarcoma. (**A**) Commonly used chemotherapeutics: cisplatin, doxorubicin, etoposide, methotrexate. (**B**–**C**) Agents with lower (**B**: dasatinib, dactolisib, entospletinib, ruxolitinib) or higher (**C**: apitolisib, afuresertib, lapatinib, tozasertib) predicted IC50 in the low-TLSPI subgroup versus high-TLSPI

Drug-sensitivity prediction was performed with oncoPredict using the GDSC database (Table S8). Among commonly used osteosarcoma chemotherapeutics (cisplatin, doxorubicin, etoposide, methotrexate), the low-TLSPI group showed lower predicted IC50 for doxorubicin (Fig. 9A). Across additional agents, the low-TLSPI group had lower predicted IC50 for dasatinib, dactolisib, entospletinib, and ruxolitinib (Fig. 9B), whereas the high-TLSPI group had lower predicted IC50 for apitolisib, afuresertib, lapatinib, and tozasertib (Fig. 9C). Collectively, these patterns suggest that TLSPI may help guide personalized therapeutic strategies in osteosarcoma.

### CXCL13 in the osteosarcoma TIME

**Fig. 10:**
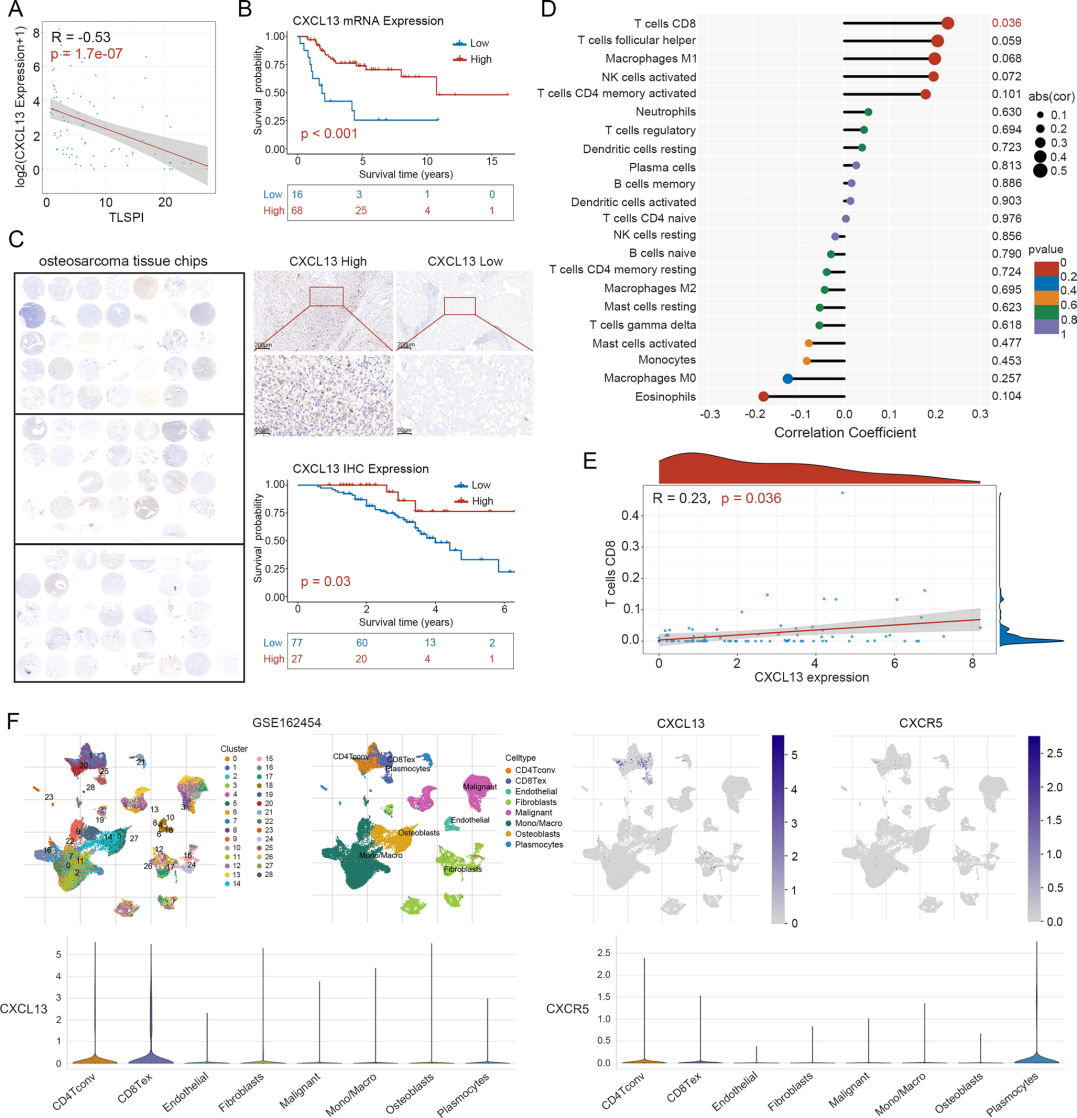
CXCL13 expression patterns and immune correlations in the osteosarcoma TIME. (**A**) Correlation between TLSPI and CXCL13 expression in the TARGET cohort. (**B**) Association between CXCL13 expression and overall survival. (**C**) Representative IHC staining of CXCL13 and KM survival analysis. (**D**) Correlation between CXCL13 expression and immune cell infiltration. (**E**) Positive association between CXCL13 expression and CD8⁺ T-cell abundance. (**F**) Single-cell UMAP visualization from the GSE162454 dataset showing CXCL13 expression in CD4⁺ Tconv and CD8⁺ Tex cells, and CXCR5 expression predominantly in plasma cells

CXCL13, one of the three genes constituting the TLSPI signature, showed marked enrichment in tumors with low TLSPI scores. In the TARGET cohort, TLSPI and CXCL13 expression were strongly inversely correlated (Fig. 10A), indicating that decreased CXCL13 levels in high-TLSPI tumors are associated with an immunosuppressive TIME. Consistently, KM analysis demonstrated that patients with low CXCL13 expression exhibited significantly poorer overall survival (Fig. 10B). IHC validation in an independent osteosarcoma cohort further confirmed these findings: tumors with low CXCL13 expression showed markedly reduced overall survival (Fig. 10C). Immune infiltration profiling revealed a positive correlation between CXCL13 expression and CD8⁺ T-cell abundance, suggesting that higher CXCL13 levels promote cytotoxic T-cell infiltration and an immune-activated phenotype (Fig. 10D–E).

Single-cell RNA sequencing data from GSE162454 analyzed via the TISCH2 platform showed that CXCL13 was predominantly expressed in CD4⁺ conventional T cells (Tconv) and CD8⁺ exhausted T cells (Tex), while its receptor CXCR5 was mainly localized to plasma cells (Fig. 10F). These findings suggest that CXCL13–CXCR5 signaling may facilitate B-cell recruitment and spatial organization within TLS, thereby fostering a more immune-permissive TIME in osteosarcoma.

### CXCL13 promotes B-cell activation, migration, and survival

**Fig. 11:**
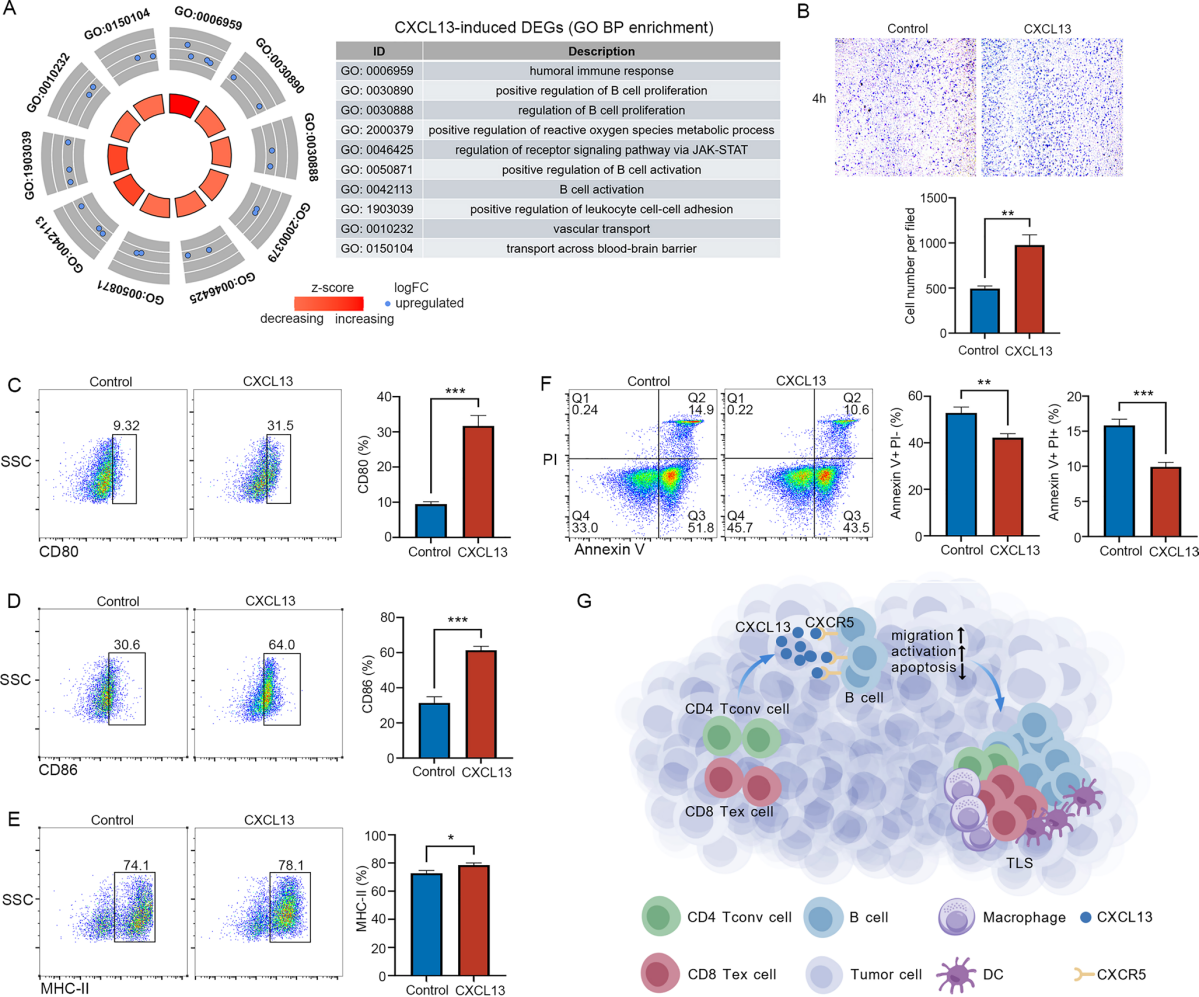
Experimental validation of CXCL13 effects on B-cell function. (**A**) GO enrichment analysis of genes upregulated in CXCL13-treated B cells. (**B**) Transwell migration assay showing CXCL13-induced B-cell chemotaxis. (**C**–**F**) Flow cytometric analysis of B-cell activation markers CD80 (**C**), CD86 (**D**), and MHC-II (**E**), as well as apoptosis indicators Annexin V and PI (**F**). (**G**) Proposed working model of CXCL13-driven TLS in osteosarcoma. Data are presented as mean ± SD; error bars indicate SD. *n* = 3 independent biological replicates

Transcriptomic profiling of CXCL13-treated B cells revealed a distinct subset of upregulated genes enriched in B-cell activation–related pathways (Fig. 11A), indicating transcriptional reprogramming toward an activated phenotype. Functional assays validated these findings: CXCL13 stimulation significantly enhanced B-cell migration in Transwell assays (Fig. 11B) and increased expression of activation markers CD80 (from 9.3% to 31.5%; Fig. 11C), CD86 (from 30.6% to 64%; Fig. 11D), and MHC-II (baseline 74.1%) showed a modest but significant increase (Fig. 11E). Furthermore, CXCL13 treatment reduced both early apoptosis (from 51.8% to 43.5%) and late apoptosis (from 14.9% to 10.6%; Fig. 11F). Collectively, these results identify CXCL13 as a pivotal modulator of B-cell activation, migration, and survival, implicating its role in TLS formation and its potential therapeutic relevance in osteosarcoma (Fig. 11G).

## Discussion

To our knowledge, this study provides the first comprehensive characterization of TLS in osteosarcoma, a malignancy historically known for its poor immunogenicity and limited responsiveness to immunotherapy [32]. Through integration of spatial immune profiling, transcriptomic modeling, and functional validation, we demonstrated that TLS are crucial components of the TIME and are significantly associated with improved prognosis. These findings establish a strong rationale for developing a TLS-related prognostic model to quantify immune activation and enhance prognostic accuracy in osteosarcoma.

Histopathological analysis identified TLS-like structures in osteosarcoma tissues, characterized by the co-localization of distinct T-cell and B-cell zones. Multiplex immunofluorescence using a validated TLS marker panel (CD3, CD4, CD8, CD20, and CD68) further demonstrated well-organized immune-cell aggregates with clear compartmentalization of lymphocyte subsets, indicative of functional TLS architecture. Importantly, the presence of these TLS within the TIME was significantly associated with improved patient prognosis, underscoring their potential as favorable prognostic indicators. Collectively, these findings highlight the pivotal role of TLS in promoting antitumor immune surveillance and shaping the immune landscape of osteosarcoma [33].

To quantify immune dynamics related to TLS, we developed TLSPI using a machine-learning framework that systematically integrated 101 modeling strategies. The final model consisted of three immune-related genes, CXCL13, IL2RA, and STAT5A, all of which are known to regulate lymphocyte recruitment, immune activation, and cytokine signaling [34–36]. TLSPI demonstrated robust and reproducible prognostic performance across multiple independent cohorts and outperformed 36 previously published osteosarcoma gene signatures, underscoring its potential as a clinically applicable biomarker for immune activation and prognosis assessment in osteosarcoma.

Consistent with previous reports in the osteosarcoma TIME [37], CIBERSORT deconvolution suggested that M0 and M2 macrophages represent the most abundant infiltrating immune cell populations, and the low-TLSPI group exhibited a higher inferred fraction of M2 macrophages. Although M2 macrophages are commonly considered pro-tumorigenic, accumulating evidence in osteosarcoma supports a context-dependent role. For example, the presence of CD163-positive, M2-polarized macrophages has been reported to be essential for restraining osteosarcoma progression, contrasting with observations in many other solid tumors [38]. Likewise, higher M2 macrophage infiltration has been associated with a more favorable prognosis in osteosarcoma [39]. Collectively, these findings underscore the heterogeneous, tumor-type–specific functions of infiltrating macrophages in osteosarcoma. In addition, low-TLSPI tumors demonstrated increased infiltration of CD8⁺ cytotoxic T cells, NK cells, and B cells, accompanied by higher stromal and immune scores. Elevated expression of immune response pathways, including CCR signaling, antigen presentation, inflammatory response, T-cell activation, and interferon response, further indicated an active, immune-inflamed state. These findings position TLSPI as a surrogate marker of a “hot” TIME characterized by robust immune surveillance and effector activity.

TLSPI was negatively correlated with the expression of multiple immune checkpoint genes, suggesting that patients with low TLSPI scores may exhibit a more active immune state and potential responsiveness to checkpoint blockade therapy. Patients with low TLSPI scores showed reduced TIDE values and higher predicted sensitivity to immune checkpoint blockade. Validation in external cohorts (GSE100797 and GSE35640) confirmed that low-TLSPI patients contained a greater proportion of responders, underscoring its translational potential as both a prognostic and predictive biomarker for immunotherapy in osteosarcoma.

Mechanistic analysis identified CXCL13 as a key regulator of TLS formation in osteosarcoma, consistent with prior findings [40, 41]. IHC analysis revealed that high CXCL13 expression correlated with favorable prognosis, while immune correlation analysis demonstrated a positive association between CXCL13 levels and CD8⁺ T-cell infiltration. Single-cell RNA sequencing revealed that CXCL13 was predominantly secreted by CD4⁺ helper T cells and exhausted CD8⁺ T cells, while its receptor CXCR5 was expressed in B-cell clusters. This CXCL13–CXCR5 axis orchestrates TLS assembly through B-cell recruitment and activation. In vitro, CXCL13 enhanced B-cell chemotaxis, upregulated activation markers (CD80, CD86, and MHC-II), and reduced apoptosis. These results indicate that CXCL13 is a pivotal mediator linking TLS organization with functional immune activation. Recent studies also suggest that CXCR5 overexpression enhances CAR-T cell trafficking and antitumor efficacy [42, 43], further supporting its therapeutic potential. These findings reinforce the role of CXCL13 in TLS biology and support its conserved function across tumor types [44–46].

Beyond immune profiling, TLSPI also revealed differential drug sensitivity. Patients with low TLSPI scores were predicted to respond better to both conventional chemotherapeutics such as doxorubicin and targeted agents including dactolisib and ruxolitinib. Conversely, high-TLSPI patients were more sensitive to agents like lapatinib and apitolisib. These results suggest that TLSPI may have utility in guiding broader treatment decisions beyond immunotherapy, providing a more personalized therapeutic roadmap for osteosarcoma patients.

Despite these promising findings, several limitations should be acknowledged. First, the retrospective nature of the bulk RNA-seq cohorts and the relatively small number of TLS-positive tumors may limit the generalizability of our conclusions. Given the limited TLS-positive cases, TLSPI should not be used to infer the presence of histological TLS structures; instead, it should be interpreted as a surrogate marker of TLS-related immune activation and lymphocyte infiltration, and spatial validation in larger cohorts is warranted. Moreover, although our in vitro experiments support a functional role for CXCL13 in B-cell activation, in vivo models are needed to validate its causal contribution to TLS formation within the osteosarcoma microenvironment. Finally, future studies should clarify whether TLS maturation status (immature vs. mature) differentially influences therapeutic outcomes.

In conclusion, this study provides the first integrative characterization of TLS in osteosarcoma and introduces a novel TLS-related prognostic model derived through a machine-learning framework. TLSPI demonstrated robust and reproducible predictive performance across multiple independent cohorts and was closely associated with immune cell infiltration, immune checkpoint expression, and potential responsiveness to immunotherapy. Mechanistic analyses identified CXCL13 as a key regulator of B-cell recruitment and activation, linking TLS organization with antitumor immune activity. By integrating transcriptomic analysis, histopathological validation, and functional assays, our findings reveal new insights into the immune architecture of osteosarcoma and establish TLSPI as a promising biomarker for guiding precision immunotherapy. These results provide a foundation for future translational studies targeting TLS-mediated immune regulation to improve outcomes in this aggressive malignancy.

## Electronic Supplementary Material

Below is the link to the electronic supplementary material.

Supplementary Material 1

Supplementary Material 2

Supplementary Material 3

Supplementary Material 4

Supplementary Material 5

## Data Availability

All public datasets enrolled in this study can be downloaded from GEO database (https://www.ncbi.nlm.nih.gov/geo/) and TARGET database (https://www.cancer.gov/ccg/research/genome-sequencing/target), or the data availability sections of the relevant publications. All data relevant to this investigation, whether generated or analyzed, are comprehensively detailed in this manuscript and its supplementary materials. For further inquiries or data requests, interested parties are advised to reach out to the corresponding authors.

## Abbreviations

TLS: Tertiary lymphoid structures
TLSPI: TLS prognostic index
ICB: Immune checkpoint blockade
CAR: Chimeric antigen receptor
MDSCs: Myeloid-derived suppressor cells
M2 TAMs: M2 tumor-associated macrophages
CAFs: Cancer-associated fibroblasts
PKUPH: Peking University People’s Hospital
FFPE: Formalin-fixed paraffin-embedded
IHC: Immunohistochemistry
mIF: Multiplex immunofluorescence
C-index: Concordance index
KM: Kaplan-Meier
tROC: Time-dependent receiver operating characteristic
DCA: Decision curve analysis
DEGs: Differentially expressed genes
GO: Gene Ontology
KEGG: Kyoto Encyclopedia of Genes and Genomes
GSEA: Gene Set Enrichment Analysis
GSVA: Gene Set Variation Analysis
TIDE: Tumor Immune Dysfunction and Exclusion
GDSC: Genomics of Drug Sensitivity in Cancer
IC50: Half-maximal inhibitory concentrations
TISCH2: Tumor Immune Single Cell Center 2
PBMCs: Peripheral blood mononuclear cells
FBS: Fetal bovine serum
HRs: Hazard ratios
SD: Standard deviation
TIME: Tumor immune microenvironment
AUC: Area under the curve
CR: Complete response
PD: Progressive disease
SD: Stable Disease
PR: Partial Response
R: Response
NR: Non-response
Tconv: Conventional T cells
Tex: Exhausted T cells
