# Supplementary figures and images for: Machine learning–based integrative analysis identifies CXCL13-driven tertiary lymphoid structures as favorable immune and prognostic features in osteosarcoma
Source: Cell Oncol (Dordr). 2026 May 16;49(4):93. doi: 10.1007/s13402-026-01226-1 (PMC13350786; doi:10.1007/s13402-026-01226-1)

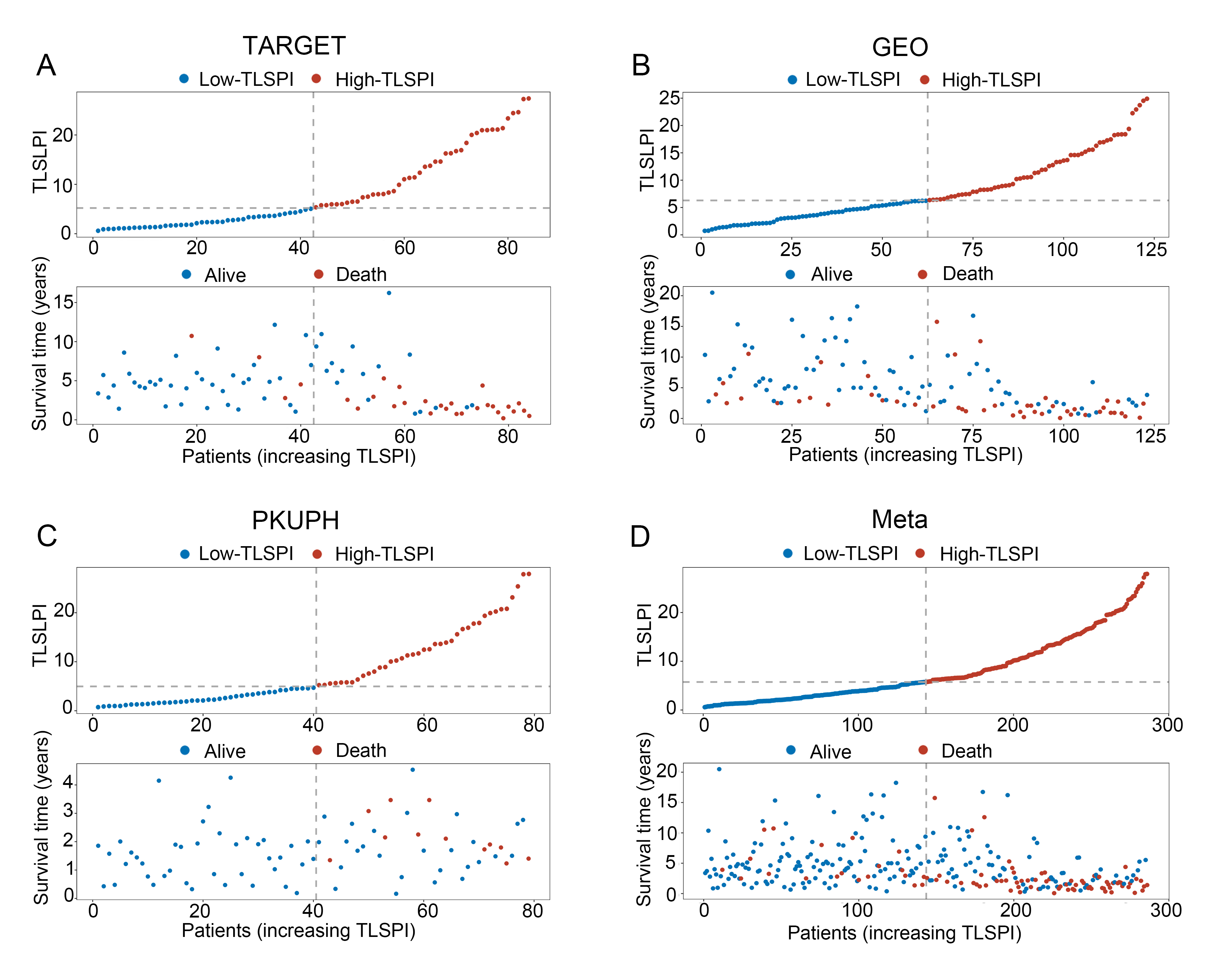

Supplement: Supplementary file 1 — Supplementary Material 1 [file 13402_2026_1226_MOESM1_ESM.jpg]

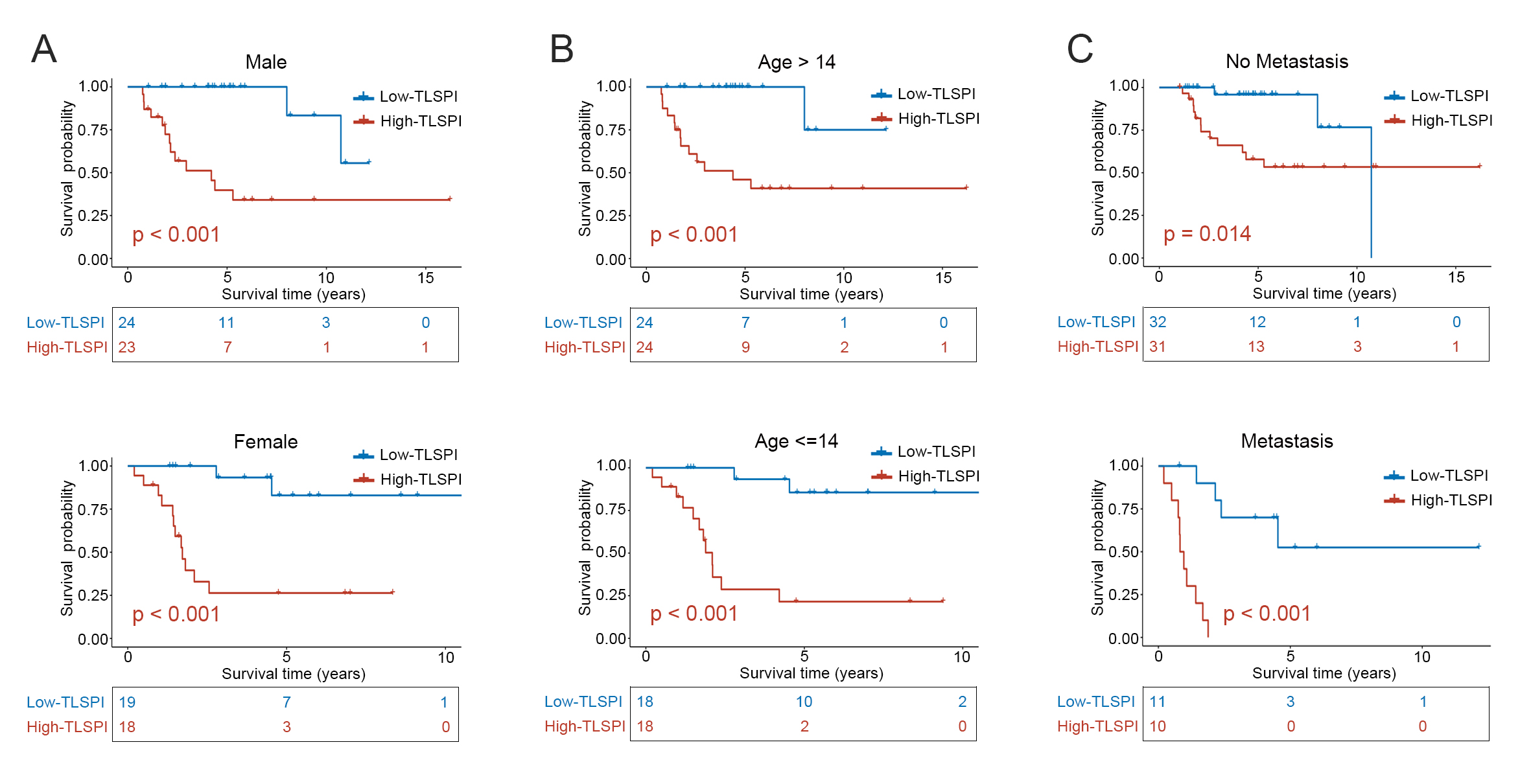

Supplement: Supplementary file 2 — Supplementary Material 2 [file 13402_2026_1226_MOESM2_ESM.jpg]
