# Supplementary material for: Machine learning–based integrative analysis identifies CXCL13-driven tertiary lymphoid structures as favorable immune and prognostic features in osteosarcoma
Source: Cell Oncol (Dordr). 2026 May 16;49(4):93. doi: 10.1007/s13402-026-01226-1 (PMC13350786; doi:10.1007/s13402-026-01226-1)
Supplement: Supplementary file 4 — Supplementary Material 4 [file 13402_2026_1226_MOESM4_ESM.docx]

**Machine learning–based integrative analysis identifies CXCL13-driven tertiary lymphoid structures as favorable immune and prognostic features in osteosarcoma**

**Jie Jiang^1^, Jiuhui Xu^1^, Lu Xie^1^, Yiyang Yu^1^, Xin Sun^1^, Xiaojiao Sun^2^, Shen Yang^2^, Huanmin Wang^2,3^, Tingting Ren^1^*, and Xiaodong Tang^1^***

1. Department of Musculoskeletal Tumor, People's Hospital, Peking University, Beijing, 100044, China

2. Department of Surgical Oncology, Beijing Children's Hospital, Capital Medical University, National Center for Children's Health, Beijing, 100045, China

3. MOE Key Laboratory of Major Diseases in Children, Beijing Children's Hospital, Capital Medical University, National Center for Children's Health, Beijing, 100045, China

*** Corresponding authors:**

**Tingting Ren**, E-mail: tumorcenter@163.com

**Xiaodong Tang,** E-mail: tang15877@163.com

This file includes: Supplemental Figures S1 to S2


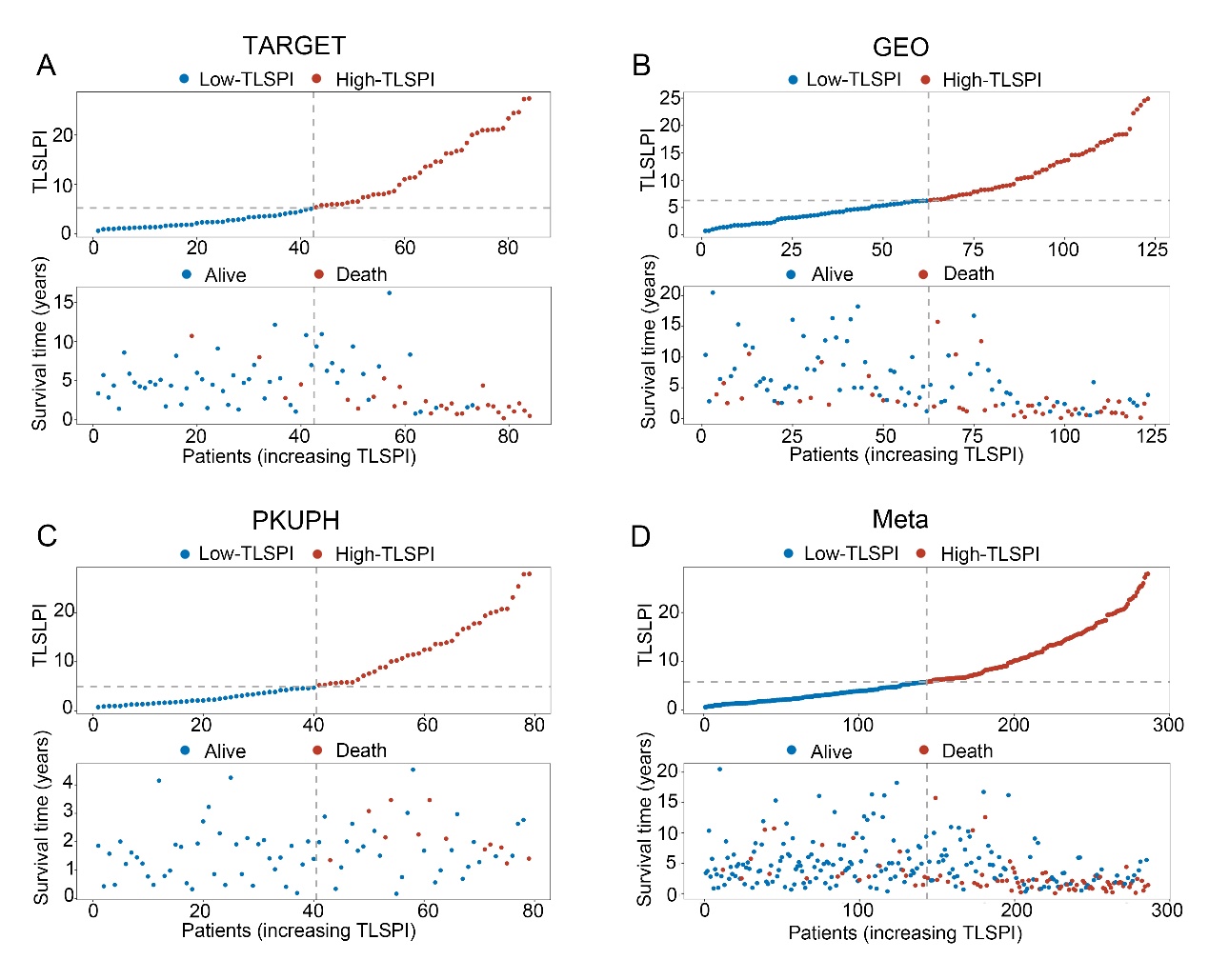


**Fig. S1.** TLSPI-based risk stratification and survival status across cohorts. In the TARGET (A), GEO (B), PKUPH (C), and Meta (D) cohorts, patients were stratified into low-TLSPI (blue) and high-TLSPI (red) subgroups based on the median TLSPI score (dashed line). Survival status is indicated by colored dots.


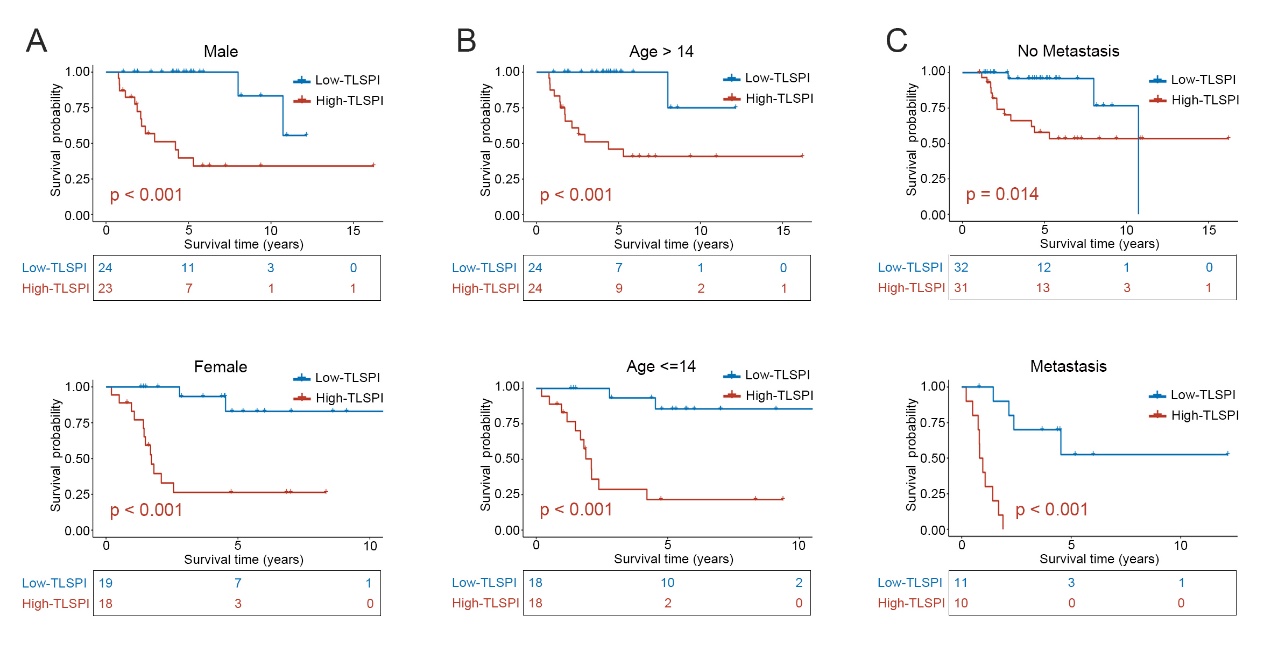


**Fig. S2.** Clinical applicability of TLSPI. KM survival analysis stratified by clinical factors including gender (A), age (B), and metastasis status (C).
